# Identification of a bet-hedging network motif generating noise in hormone concentrations and germination propensity in *Arabidopsis*

**DOI:** 10.1098/rsif.2018.0042

**Published:** 2018-04-11

**Authors:** Iain G. Johnston, George W. Bassel

**Affiliations:** School of Biosciences, University of Birmingham, Birmingham, UK

**Keywords:** plant hormones, germination, cell noise, stochastic processes, food security, bet-hedging

## Abstract

Plants have evolved to exploit stochasticity to hedge bets and ensure robustness to varying environments between generations. In agriculture, environments are more controlled, and this evolved variability decreases potential yields, posing agronomic and food security challenges. Understanding how plant cells generate and harness noise thus presents options for engineering more uniform crop performance. Here, we use stochastic chemical kinetic modelling to analyse a hormone feedback signalling motif in *Arabidopsis thaliana* seeds that can generate tunable levels of noise in the hormone ABA, governing germination propensity. The key feature of the motif is simultaneous positive feedback regulation of both ABA production and degradation pathways, allowing tunable noise while retaining a constant mean level. We uncover surprisingly rich behaviour underlying the control of levels of, and noise in, ABA abundance. We obtain approximate analytic solutions for steady-state hormone level means and variances under general conditions, showing that antagonistic self-promoting and self-repressing interactions can together be tuned to induce noise while preserving mean hormone levels. We compare different potential architectures for this ‘random output generator’ with the motif found in *Arabidopsis*, and report the requirements for tunable control of noise in each case. We identify interventions that may facilitate large decreases in variability in germination propensity, in particular, the turnover of signalling intermediates and the sensitivity of synthesis and degradation machinery, as potentially valuable crop engineering targets.

## 1. Introduction

Stochasticity is an unavoidable feature underlying cell biology [1–3], with random influences affecting a multitude of processes in cells [4–8]. Classic examples of processes where noise plays a central role in determining biological behaviour include gene expression [4,7–12], stem cell fate decisions [13], cancer development [14,15] and organelle population dynamics [16–19]. Theoretical work has often focused on how cellular circuitry can provide robustness to intrinsic noise [10,20,21], with comparatively little emphasis on how biology may exploit intrinsic noise to generate useful structure or variation [22]. One well-known example of such exploitation is bet-hedging in bacterial phenotypes, where variability within a population is used to provide robustness to potentially varying environments [23,24].

The biology of seeds provides an agriculturally vital example of eukaryotic noise exploitation [25,26]. Plants are sessile organisms and cannot readily move away from challenging environments. An evolutionary priority for plants is to ensure that future generations survive in the face of environmental change [27–29]. To this end, plants induce and exploit variability between seed responses to the environment to hedge against different conditions [25,28], leading to, for example, differences in germination propensity between seeds [30,31]. This variability is evolutionarily beneficial—for example, seed-to-seed variability in germination propensity may allow a subset of seeds to remain dormant and survive through an environmental challenge, while seedlings from early-germinating seeds perish. However, in agricultural circumstances, the environments that plants face are more controlled and often less challenging than the past ecological environments they have evolved to hedge against. The induction of seed-to-seed variability is then no longer beneficial, and instead poses agronomic challenges, such as preventing uniform establishment of field crops [25,30,32]. Clearly, in such circumstances, artificial interventions to mitigate the evolved mechanisms generating variability are desirable. Understanding the mechanistic basis of these noise-inducing processes will thus allow the engineering of plants with more homogeneous traits of human interest, including germination propensity.

Variability in biological systems can be separated into that arising from so-called extrinsic and intrinsic sources [25]. Extrinsic variability arises externally from an individual's environment, while intrinsic variability is generated within an individual (individuals here may be, for example, cells, seeds or organisms, depending on the scale of study). Extrinsic variability has been demonstrated to impact plant strategies [26], including the extent of seed dormancy based on the maternal environment in which seeds develop; this dependence has been studied extensively previously [33,34]. Intrinsic variability is also present within seeds [25,27], but remains unexplored, despite the central importance of seed variability to science and world agriculture.

Here, we report and analyse an intrinsic noise-generating network motif observed in the metabolic circuit governing germination decisions in the model plant *Arabidopsis thaliana*. This motif, consisting of a coupled self-promotion and self-repressing pathway, functions as a ‘random output generator’, allowing the tunable induction of noise in levels of abscisic acid (ABA), a hormone that represses germination. Three-fold variability in levels of ABA in seeds from the same silique in *Arabidopsis* has been observed [31], pointing to the induction of ABA variability as a controlled route to bet-hedging in plant seed production.

Using tools from stochastic processes and simulation, we derive an analytical description of noise induction in this system and elucidate its dependence on biological features that are susceptible to artificial engineering. We thus use this stochastic modelling approach to suggest synthetic manipulation strategies to decrease noise in germination propensity and address the associated agronomic issues.

### 1.1. An antagonistic feedback system in *Arabidopsis*

**Figure 1.**
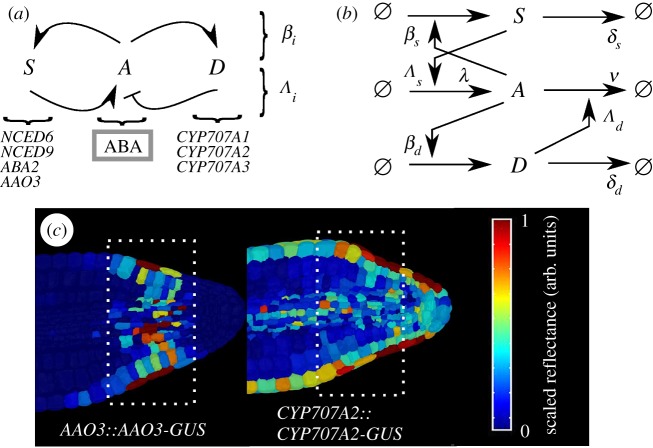
An antagonistic pair of feedback loops governs hormone levels in *Arabidopsis*. (*a*) Synthesis and degradation of the hormone ABA (species *A*) are modulated by two pathways (involving *S* and *D*, respectively) that themselves respond to ABA levels. Throughout this work, we will use parameters *β*_*i*_ to correspond to the strengths of these responses, and *Λ*_*i*_ to correspond to the sensitivity of ABA to these signals. (*b*) *A*, *S* and *D* are produced and degraded, with levels modulating the rates of these processes. (*c*) Fluorescence microscopy following Topham *et al*. [37] identifies cellular localization of members of the *S* and *D* pathways in *Arabidopsis* embryos, showing that they are both present (scaled reflectance >0) in a range of cells (highlighted) at the same developmental stage. The antagonistic feedback loops thus together modulate ABA levels in these cells.

We first introduce the recent experimental characterization of the feedback architecture that we will study. The hormone ABA plays a central role in a set of metabolic interactions in plant cells that determine germination behaviour [35]. In *Arabidopsis* seeds, ABA is synthesized by a metabolic pathway involving *NCED6*, *NCED9*, *ABA2* and *AAO3* and degraded by *CYP707A1*, *CYP707A2* and *CYP707A3* [36,37]. The response to ABA induces upregulation of its synthesis genes and induces upregulation of *CYP707A2*, the major contributor to ABA breakdown in seeds, in its degradation pathway [37]. The system therefore consists of a metabolic feedback system inducing ABA synthesis and ABA degradation, both upregulated by ABA responses, schematically illustrated in figure 1*a*, with specific processes labelled in figure 1*b*.

This circuit exists as part of a wider regulatory network involving the hormone gibberellic acid (GA), which interacts antagonistically with ABA [38,39] and promotes germination. Previous analysis of the plant embryo [37] has shown that cells dominated by ABA responses and those dominated by GA responses are spatially separated in *Arabidopsis*. Levels of GA and stochastic influences in the wider signalling architecture represent further potential sources of variability [40], but we hypothesized that the ABA-centric motif alone may be sufficient to generate appreciable noise in hormone levels and downstream behaviour. We, therefore, focus on the subset of this wider network that is centred on ABA response and its feedbacks onto ABA synthesis and degradation, to explore the intrinsic dynamics of this hormone and directly related signalling pathways.

## 2. Results

Fluorescence microscopy experiments reveal that elements of the synthesis and degradation pathways are present in the same cells in the *Arabidopsis* embryo (electronic supplementary material; figure 1*c*). This observation is reproducibly made in embryos imaged at the same developmental stage, and points to the concurrent presence of these antagonistic elements in cells [37]. This concurrency suggests that both pathways may be active in controlling ABA in a given cell; a scenario supported by previous modelling work [37], which successfully described and predicted germination behaviour assuming the presence of this antagonistic network motif.

If it was only mean hormone levels that had a functional role in dictating cellular behaviour, this joint expression may be viewed as unnecessarily inefficient: any desired positive or negative change in the mean level could presumably be achieved through one pathway alone. However, we hypothesized that this antagonistic activity facilitates the control of *variability* independent of the mean level. In the light of bet-hedging strategies in plant evolution [25,28], we thus sought to explore how these antagonistic pathways may act together to induce a controllable level of noise in ABA, and hence provide a tunable ‘random output generator’ underlying the germination decision.

### 2.1. The stochastic behaviour of the feedback motif

We use a stochastic chemical kinetic framework to describe a system where a central chemical species *A* (representing ABA) evolves in conjunction with a species *S* that promotes its synthesis and a species *D* that promotes its degradation. We coarse-grain the processes of chemical synthesis and degradation into Poissonian immigration and death terms, respectively, and model the transduction of each independent signal as occurring through pathways involving a single intermediate. We note that the genes and enzymes underlying these pathways are known [36,37] and subject to stochastic chemical kinetics in their own production and degradation, but the straightforward structure of these pathways motivates this coarse-graining to allow a more intuitive understanding of the system's behaviour. We will initially work in the picture of interactions taking place in a single cell.

As in figure 1, *A* catalyses the synthesis of *S* and *D*, which, respectively, increase the rates of *A* synthesis and degradation. *S* and *D* themselves degrade according to a Poissonian death term. The overall model is thus
2.1
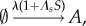

2.2
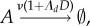

2.3
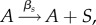

2.4
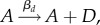

2.5
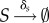

2.6
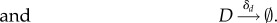
*β* parameters denote the strength with which *A* promotes the production of *S* and *D* and can be viewed as the cell's responsiveness to *A* levels. *Λ* parameters control the strength with which *S* and *D* influence the synthesis and degradation of *A*; λ and *ν* are the base synthesis and degradation rates of *A*; *δ* parameters are the degradation rates of the two feedback species. All parameters describe rates, and are throughout taken to be unitless multiples of a characteristic time scale *τ*. The system is illustrated in figure 1*b*.

**Figure 2.**
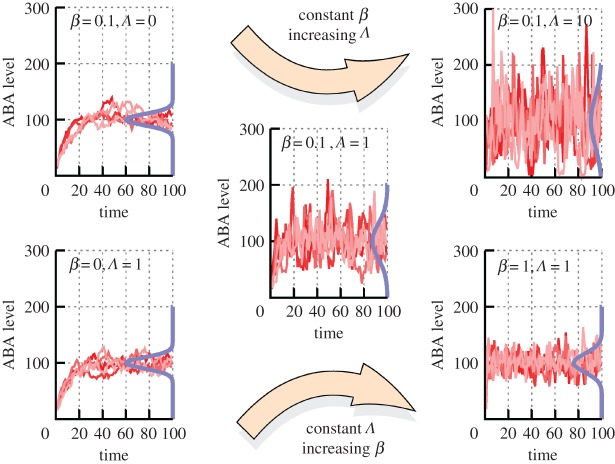
Hormone levels over time as a function of feedback responses and sensitivities. Behaviour of hormone level *A* over time, in five stochastic simulations in each panel, as (top) sensitivity *Λ* and (bottom) response *β* change. Increasing *Λ* increases noise monotonically while preserving the mean hormone level; increasing *β* drives noise levels through a peak before decreasing. (Online version in colour.)

We first consider the symmetric realization of this system with *Λ*_*s*_ = *Λ*_*d*_ = *Λ*, *β*_*s*_ = *β*_*d*_ = *β* and *δ*_*s*_ = *δ*_*d*_ = *δ*. This symmetric case corresponds to each type of biological process having the same rate, which we regard as a simple ‘default’ case; we will generalize this picture later. We use stochastic simulation to investigate the induction of noise through this antagonistic mechanism with an example set of parameters *β* = 0.1, *δ* = 1, λ = 10, *ν* = 0.1 (results for general parameters will be derived later). In the absence of sensitivity (*Λ* = 0), the numerical results converge on the well-known results for an immigration–death process *ϕ*_*A*_ = λ/*ν*, 〈*ξ*^2^_*A*_〉 = λ/*ν* (figure 2). The noise, expressed as a coefficient of variation, is thus 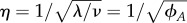, in agreement with the common 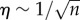 scaling seen in other biological contexts [12]. As *Λ* increases, the level of noise increases from this base case to several-fold higher, while the mean level is preserved as λ/*ν* (figure 2). Increasing the sensitivity of hormone synthesis and degradation to the presence of the intermediate signalling molecules—in essence, increasing the strength of the feedback signal—thus increases the noise in hormone level while keeping *ϕ*_*A*_ constant.

As *β* increases from zero, the level of noise increases to a peak, then, by contrast, subsequently decreases (figure 2). Increasing the response of the hormone synthesis and degradation machinery to hormone levels thus induces non-monotonic behaviour in noise. This non-monotonic behaviour can be understood as resulting from a tension between the strength of the response of *S* and *D* intermediates to *A* levels (requiring a high *β*) and the variability resulting from the dynamics of *S* and *D* (increased at low copy number and thus low *β*). Once more, tuning *β* modulates noise in *A* without affecting the mean level of *A*.

Intuitively, in this symmetric case, the synthesis and degradation signals remain of the same average magnitude, so the average hormone level remains the same. But due to intrinsic fluctuations in the levels of the signalling molecules, higher dependence of *A* on the dynamics of these molecules leads to higher variability in *A*. Observing that figure 2 suggests that the system converges to a steady-state distribution for a variety of parametrizations, we proceed by attempting to find interpretable expressions for the properties of this steady-state behaviour.

### 2.2. Analysis of the symmetric feedback system

As is often the case with chemical kinetic models, this system cannot be readily solved to yield exact analytic solutions for the behaviour of interest. However, we can employ a linear noise approximation via Van Kampen's system size expansion [41,42] to characterize the levels of induced noise. Briefly, this approximation involves representing the level of chemical species in a system as a sum of a deterministic component *ϕ* and a fluctuating component *ξ* (both vectors with *n*_*s*_ components), interpreted, respectively, as encoding the mean and random behaviours of the *n*_*s*_ species in the system. As *ξ* has zero mean (mean species levels being encoded by *ϕ*), 〈*ξ*_*i*_〉^2^ = 0, and 〈*ξ*^2^_*i*_〉 is interpreted as the variance associated with the level of species *i*. The chemical master equation is then phrased in terms of these elements, and we collect terms that scale in different powers of the system size. If the system of interest involving *R* reactions is represented by a stoichiometric matrix *S* and a vector of rates **f**, this process gives us (see the electronic supplementary material) a set of ODEs describing the system's mean behaviour *ϕ* and a Fokker–Planck equation describing the behaviour of the fluctuating components. The 3 × 6 stochiometric matrix *S* and 1 × 6 vector **f** of rates for our system are readily written down from equations (2.1)–(2.6), then we obtain ODEs for the mean behaviour (see the electronic supplementary material) which support the steady-state solution suggested by numeric simulation above. Specifically, if *ϕ*_*i*_ denotes the mean level of species *i*, we obtain
2.7
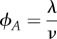
and
2.8
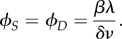
We also obtain ODEs (see the electronic supplementary material) for the variances and covariances of the fluctuating components which can be solved in the steady state, giving in particular a solution for the variance of *A*:
2.9
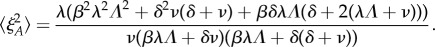
Equation (2.9) allows us to explore how the variance, and noise (
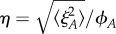
, which for constant *ϕ*_*A*_ here gives 
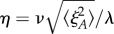
) of hormone levels change with the strength of the feedback signals mediated via *S* and *D*. When *Λ* = 0 (*A* is insensitive to *S* and *D*), this reduces to the expected 〈*ξ*^2^_*A*_〉 = λ/*ν* (hence 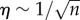 as above) for a simple immigration–death process governing ABA dynamics. Taking derivatives shows that as *Λ* rises, 〈*ξ*^2^_*A*_〉 and *η* undergo a monotonic increase to a saturating value. The maximum variance is thus achieved as 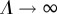, giving
2.10
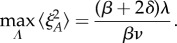
The variance—and the Fano factor—of ABA levels thus spans a multiplicative range of (*β* + 2*δ*)/*β* as *Λ* increases from 0; the coefficient of variation *η* spans a multiplicative range of 
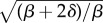
 from its 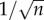 base case. Hence, the relationship *δ*/*β* between the degradation and synthesis rates of the intermediate species plays a crucial role in determining the degree to which hormone noise can be manipulated by tuning sensitivity.

**Figure 3.**
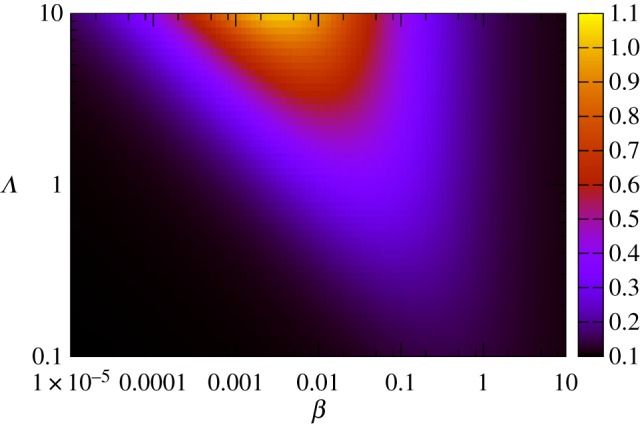
Noise in hormone levels as a function of *β* and *Λ*. Noise *η* in hormone levels predicted by equation (2.9) as sensitivity *Λ* and response *β* change over orders of magnitude. As suggested by figure 2 and shown by equation (2.9), increasing *Λ* monotonically increases *η* for a given *β* (saturating according to equation (2.10), and increasing *β* drives *η* through a peak (equation (2.11)). Mean hormone levels are constant at λ/*ν* throughout this phase plane. (Online version in colour.)

As seen in simulations above (figure 2), the behaviour of 〈*ξ*^2^_*A*_〉 and *η* with *β* for a given *Λ* is not monotonic. When *β* = 0 (synthesis of *S* and *D* is not catalysed by *A*), 〈*ξ*^2^_*A*_〉 again reduces to λ/*ν*, and when 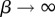, 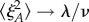. Computing *d*〈*ξ*^2^_*A*_〉/*dβ* shows that, as *β* rises, 〈*ξ*^2^_*A*_〉 rapidly rises to a peak at 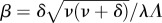, at which 〈*ξ*^2^_*A*_〉 takes the value
2.11
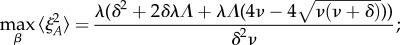
the maximum value of 〈*ξ*^2^_*A*_〉 achievable by tuning *β* is thus strongly dependent on sensitivity *Λ*. Figure 3 shows the structure of *η* behaviour as *β* and *Λ* are tuned. We underline that through these changes to *β* and *Λ*, the mean hormone level *ϕ*_*A*_ remains constant at *ϕ*_*A*_ = λ/*ν*.

Here and throughout, we confirmed that the analytic predictions from the linear noise approximation matched the behaviour of numerical simulations. The agreement between theory and simulation is strong for all parametrizations considered (see the electronic supplementary material).

### 2.3. Generalization to asymmetric regulatory interactions

We have shown that symmetric feedback strengths (*β*_*s*_ = *β*_*d*_, *Λ*_*s*_ = *Λ*_*d*_) provide the plant cell with a robust way of modulating noise while retaining the mean level of a hormone. It is of interest to generalize these results to the case of asymmetric interactions, both to better capture potential heterogeneity in interaction strengths that may occur in biology, and to explore the effects of synthetic interventions to change individual features of the symmetric regulatory system.

A steady-state solution for all means, variances and covariances in the case of different *β*_*s*_, *β*_*d*_, *δ*_*s*_, *δ*_*d*_, *Λ*_*s*_, *Λ*_*d*_ can readily be found by applying the above treatment to the general equations (2.1)–(2.6), but the form of 〈*ξ*^2^_*A*_〉 is rather lengthy and does not admit intuitive interpretation. A more informative result can be found without sacrificing much generality by setting *δ*_*s*_ = *δ*_*d*_ = 1 (hence, synthesis and degradation intermediates are degraded at the same rate, by which the rates of all other processes are scaled). Changes in these *δ* parameters have intuitive effects on the mean and variability behaviour of the system (see the electronic supplementary material). We then find, again for the steady state, that
2.12
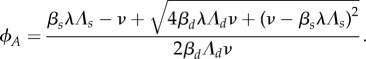
Clearly, when *β*_*s*_ ≠ *β*_*d*_ or *Λ*_*s*_ ≠ *Λ*_*d*_, *ϕ*_*A*_ departs from its usual value of λ/*ν*: a signal of synthesis or degradation being favoured by the system, and a consequent raising or lowering of steady-state mean expression.

An expression for the variance can also be derived:


2.13
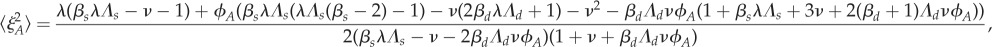


demonstrating the strongly coupled roles of the *β* and *Λ* parameters in dictating the statistics of hormone levels, and suggesting that two forms of intervention—altering sensitivity or expression levels of intermediates—can be used to artificially tune variability.

**Figure 4.**
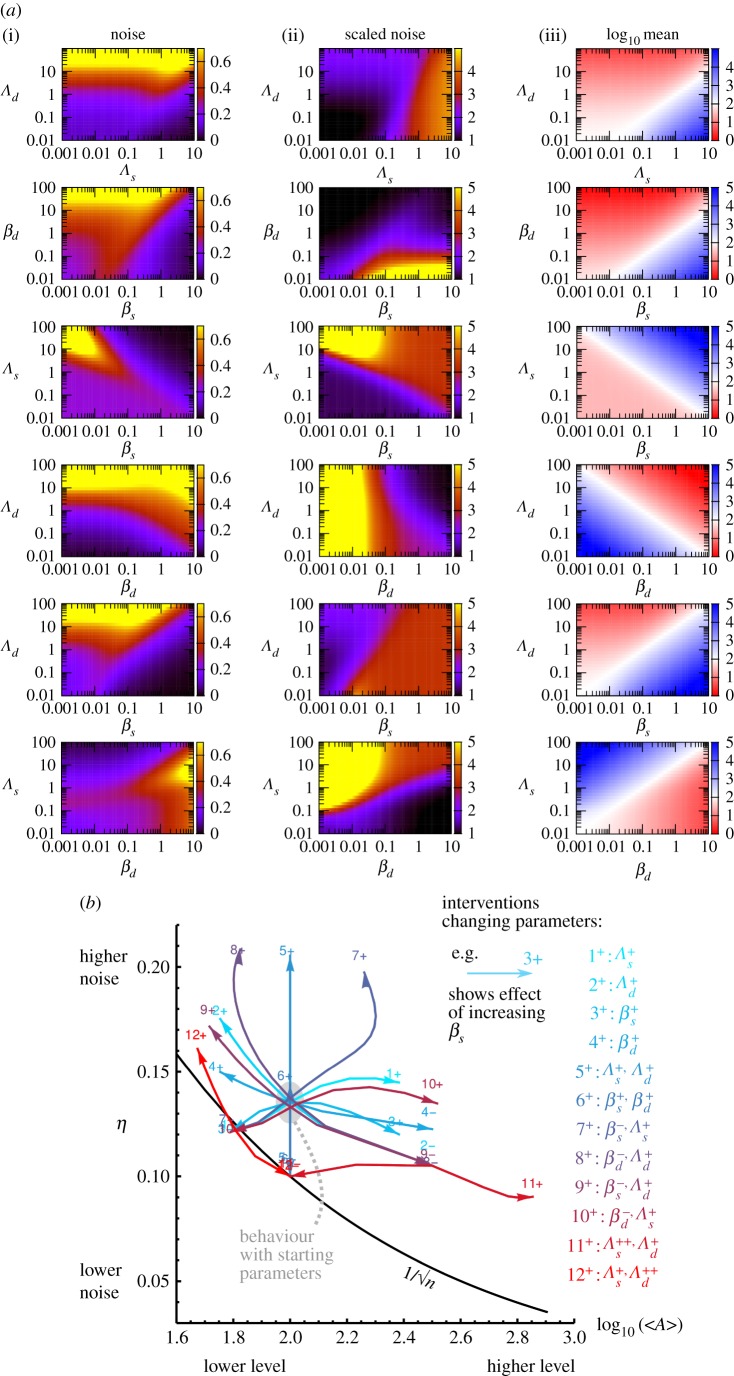
A general solution for noise under asymmetric interactions provides a roadmap for artificial interventions. (*a*) Phase portraits for the asymmetric model: behaviour of (i) noise *η*, (ii) scaled noise *η*′ and (iii) logarithm of mean level *ϕ*_*A*_ as pairs of parameters are varied. For *ϕ*_*A*_, colours denote increases (blue) and decreases (red) from the usual λ/*ν* value. (*b*) Predicted response of mean hormone level *ϕ*_*A*_ and noise *η* for a variety of interventions. Each arrow shows the resultant motion in the (*ϕ*_*A*_, *η*) plane when, starting from the default initial parametrization (corresponding to the grey ellipse), some parameters are increased or decreased according to the legend. The black line shows 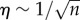.

Equations (2.12) and (2.13) together provide a predictive ‘roadmap’ for the influence of perturbed interactions on the statistics of hormone levels in the system. The behaviour of these predicted statistics under changes to each parameter is illustrated in figure 4*a*, where we use a default set of parameters (as above) with *β*_*s,d*_ = *Λ*_*s,d*_ = 0.1, *δ* = 1, λ = 10, *ν* = 0.1, and vary pairs of values while holding the remainder constant. Generally, the behaviour of mean hormone level *ϕ*_*A*_ behaves intuitively with *Λ* and *β* parameters. As *Λ*_*s*_ and *β*_*s*_ increase, *ϕ*_*A*_ increases; as *Λ*_*s*_ and *β*_*s*_ decrease, *ϕ*_*A*_ decreases to a minimum of λ/*ν*. As *Λ*_*d*_ and *β*_*d*_ increase, *ϕ*_*A*_ decreases—no longer bounded by λ/*ν*—and as *Λ*_*d*_ and *β*_*d*_ decrease, *ϕ*_*A*_ increases.

The behaviour of *η* with these control parameters is more complex and now frequently non-monotonic. Increasing *Λ*_*s*_ independently of other parameters can drive *η* to and past a maximum noise value; this non-monotonic behaviour is observed at low *β*_*s*_ and high *β*_*d*_. Similarly, increasing *β*_*s*_ independently drives *η* through and past a peak. Increasing *Λ*_*d*_ generally induces an increase in *η*. Increasing *β*_*d*_ induces a wide range of behaviours, including an increase in *η* at high *Λ*_*s*_, and a decrease followed by recovery of *η* at high *Λ*_*d*_.

This complex behaviour can be more readily interpreted by considering the quantity *η*′ ≡ *η*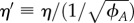, the multiplicative factor by which *η* exceeds the 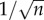 level expected for a simple immigration–death model. *η*′ reflects the additional contribution of feedback to the natural noise in hormone levels. The behaviour of this ‘scaled noise’ *η*′ with interventions is more intuitive, roughly following a ‘more synthesis, more scaled noise’ principle. Increasing *β*_*d*_ decreases *η*′; increasing *Λ*_*d*_ decreases *η*′; increasing *Λ*_*s*_ increases *η*′; increasing *β*_*s*_ increases *η*′ at low *Λ*_*s*_ and drives *η*′ through a peak at high *Λ*_*s*_. Efficient noise reduction can be achieved by reducing *β*_*s*_ and *Λ*_*s*_ or increasing *β*_*d*_ and decreasing *Λ*_*s*_.

### 2.4. Artificial interventions to modulate noise in seed behaviour

The many directions in which equations (2.12) and (2.13) show that noise can be modulated suggest a wide range of options for tuning noise in the hormone regulatory system. Figure 4*b* illustrates the effect of a set of different perturbations on *ϕ*_*A*_ and *η*. From an intermediate initial state, any combination of increasing, maintaining or decreasing *ϕ*_*A*_ and increasing, maintaining or decreasing *η* is possible by selecting the corresponding parameter(s) and directional change(s) from figure 4*b*. Perhaps the most agriculturally pertinent outcomes involve (a) decreasing noise while maintaining expression levels and (b) decreasing noise while decreasing expression levels (hence both favouring and harmonizing germination propensity). These goals can be achieved by (a) simultaneously decreasing sensitivities to, or responses of, both pathways, as seen above (*Λ*^−^_*s*_, *Λ*^−^_*d*_ and *β*^−^_*s*_, *β*^−^_*d*_) and (b) decreasing *β*_*s*_ and/or *Λ*_*s*_, increasing *β*_*s*_ while decreasing *Λ*_*s*_ or increasing *β*_*d*_ while decreasing *Λ*_*s*_.

Notably, it is possible to decrease *η* below the 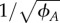 behaviour expected from the underlying immigration–death process, by strengthening the negative feedback aspects of the regulatory system. In the limit 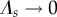 (the limit 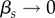 behaves equivalently, as the two parameters always appear together), negative feedback dominates (*A* represses its own production), acting to stabilize expression levels and decrease noise. In this limit, writing 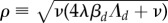,
2.14
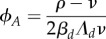
and
2.15
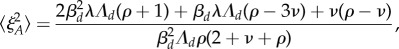
driving *η*′ below one for some parametrizations as seen in figure 4*b* (the mean-and-noise strategies listed above, and increasing *Λ*_*d*_ to a greater degree than *Λ_s_*).

As above, we confirmed that this theory matches stochastic simulation by comparing results for *ϕ*_*A*_ and 〈*ξ*^2^_*A*_〉 at uniformly spaced points in parameter space through each of the panels in figure 4*a*; the results show strong agreement and are illustrated in the electronic supplementary material.

### 2.5. Experimental data

Our model makes predictions about how perturbing aspects of the signalling circuitry in figure 1 will influence hormone levels and noise in those levels. Experimental exploration of perturbations to this motif is currently limited (though experimental evidence for substantial variability in ABA between seeds is well established, including the aforementioned threefold variability in levels of ABA in seeds from the same *Arabidopsis* silique [31]). However, one experimental study [43] artificially introduced a positive feedback circuit into *Arabidopsis*, enhancing ABA response-mediated synthesis, and providing the opportunity to test the predictions of this theory. In several plant lines including this modification, seed-wide ABA levels and germination propensity were reported for the mutated plant line and a wild-type control.

**Figure 5.**
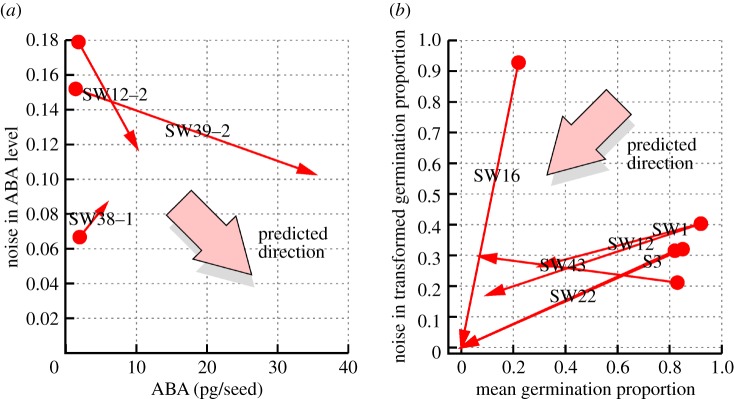
Experimental results of increasing *β*_*s*_. Arrows denote the change in behaviour from wild-type *Arabidopsis* to a mutant line where a transgenically introduced positive feedback circuit enhances the hormone synthesis pathway via *β*_*s*_. (*a*) Absolute ABA levels are generally both increased and harmonized with *β*_*s*_. (*b*) Germination propensity (inversely linked to ABA) generally decreases with *β*_*s*_, with noise in germination propensity also decreasing with *β*_*s*_, in line with higher, more harmonized ABA levels. (Online version in colour.)

We first consider the three lines where seed-wide ABA levels were reported for controls and engineered lines enhancing ABA synthesis machinery (hence, increasing *β*_*s*_ in our nomenclature). As these statistics are seed-wide, we must consider the whole-seed statistics *ϕ*^(seed)^_*A*_ and *η*^(seed)^, which reflect but are not directly linked to the microscopic *ϕ*_*A*_ and *η* on smaller (cellular) length scales (see Discussion). In each experimental case, *ϕ*^(seed)^_*A*_ was unsurprisingly increased by the increase in *β*_*s*_. We also observed that noise *η*^(seed)^ in ABA levels was markedly decreased by the genetic intervention, agreeing with our theory, in two cases, displaying a small increase in the third (figure 5*a*).

The relationship between ABA levels and germination propensity is complicated by the presence of other factors which may vary between seeds, including levels of GA, the antagonistic germination-promoting hormone. In the absence of heterogeneity in external factors, and for a simple inverse relationship between ABA level and germination propensity, we would expect decreases in germination to be a signal of increased ABA levels, and hence lower noise in ABA and germination. Using a logit transformation to cast germination percentages onto the full real line (hence accounting for the 0% and 100% boundaries in percentage statistics; see the electronic supplementary material), we found that 5 of 6 tractable experiments showed a decrease in noise in transformed germination propensity in the ABA-enhanced mutant, agreeing with our extrapolated theory (figure 5*b*).

These limited available experimental observations agree with our theory but are certainly not conclusive evidence that our model is correct. Further work inducing perturbations to the regulatory system will be required to provide stronger support for, and more power to parametrize, our model (see Discussion).

### 2.6. Alternative regulatory architectures

For completeness, we consider two alternative motifs allowing hormone levels to leverage control over hormone synthesis and degradation, involving ‘direct feedback’:
2.16
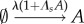
and
2.17
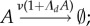
and ‘single pathway’:
2.18
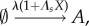

2.19
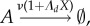

2.20
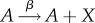

**Figure 6.**
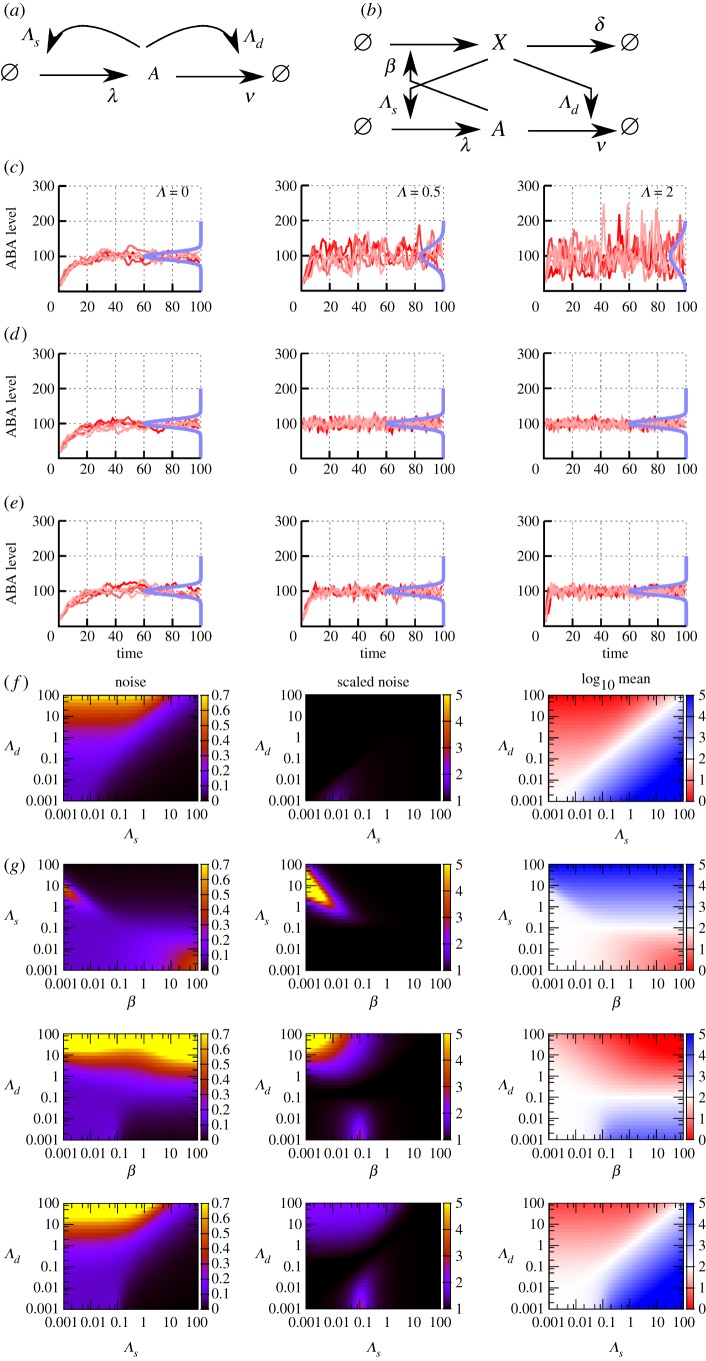
Behaviour of alternative regulatory motifs with similar structure. (*a*) The ‘direct feedback’ model, where levels of *A* directly regulate the production and degradation of *A*. (*b*) The ‘single-pathway’ model, where a single species *X* responds to *A* levels and influences both synthesis and degradation of *A*. (*c*–*e*) *Two pathways are required for noise induction through symmetric regulation.* Traces of hormone levels in five stochastic simulations, as in figure 2, for increasing *Λ*. The two-pathway system (*c*) allows increasing *Λ* to induce noise as above; the single-pathway (*d*) and direct feedback (*e*) systems show no increased noise with *Λ*. (*f*,*g*) *Behaviour of hormone levels under alternative regulatory models.* (*f*) Noise *η*, scaled noise *η*′ and logarithm of mean level *ϕ*_*A*_ as *Λ*_*s*_ and *Λ*_*d*_ vary in the ‘direct feedback’ model. Negative feedback alone can drive noise below its usual λ/*ν* level. (*g*) Noise, scaled noise and mean level for parameter changes in the ‘single-pathway’ model.

2.21
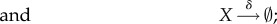
illustrated in figure 6.

For direct feedback, *A* levels directly modulate the synthesis and degradation rates of *A*. For single pathway, *A* levels modulate the synthesis of a single chemical species *X*, levels of which modulate synthesis and degradation of *A*.

To investigate whether these simpler architectures are capable of inducing the striking symmetric control over hormone levels seen previously—allowing a tuning of noise while retaining the same mean level—we first consider the case of symmetric sensitivities *Λ*_*s*_ = *Λ*_*d*_ = *Λ*. We proceed through the same analysis as above (noting that the direct feedback model admits full solutions to the equations of motion). In both cases, the steady-state solutions are
2.22
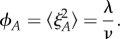


Hence, for symmetric regulatory interactions in these models, the value of *Λ* (and those of *β*, *δ*) exerts no regulatory control on the noise, which remains at the usual 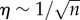 (figure 6). Indeed, *Λ*, *β*, *δ* do not control the mean levels of *A* in these symmetric cases. To control the level of noise with symmetric regulatory interactions, the two pathways *S* and *D* are required.

We can also consider the effect of varying interactions asymmetrically under these other regulatory models. In the direct feedback model we obtain
2.23
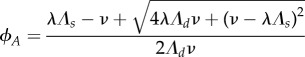
and
2.24
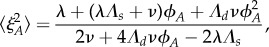
with *η*, *η*′ and *ϕ*_*A*_ behaviour shown in figure 6. As above, *ϕ*_*A*_ varies intuitively with signal strength, increasing as *Λ*_*s*_ increases and decreasing as *Λ*_*d*_ increases. Unscaled noise *η* increases with *Λ*_*d*_ and decreases with *Λ*_*s*_, but due to changes in *ϕ*_*A*_, the difference between this noise level and the expected 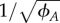 scaling is more nuanced. With increasing *Λ*_*s*_, *η*′ tends to a saturating value: at low *Λ*_*d*_ this increase drives *η*′ through a peak. When *Λ*_*d*_ ≫ *Λ*_*s*_ in this system (the negative-feedback-only case), noise is reduced: in the limit of 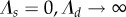, 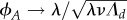 and 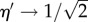, allowing control of noise below the *η*′ = 1 level expected for the simple immigration–death system. Hence, increasing *Λ*_*s*_ is optimal for decreasing absolute noise levels; increasing *Λ*_*d*_ increases absolute noise levels but is the optimal strategy for decreasing scaled noise *η*′.

The single-pathway system has very similar expressions for hormone statistics to that for the two-pathway system with *β*_*s*_ = *β*_*d*_ = *β*. Indeed, *ϕ*_*A*_ is identical to equation (2.12) in this case. A subtle but important difference exists in the expressions for 〈*ξ*^2^_*A*_〉 in the two cases (see the electronic supplementary material). As noted above, the one-pathway version results in 〈*ξ*^2^_*A*_〉 reducing to λ/*ν* when *Λ*_*s*_ = *Λ*_*d*_, removing the ability to control noise through symmetric interactions. The range of behaviours in *η* and *η*′ that can be induced by varying parameters is correspondingly altered. As *β* now represents a general response term, its influence on noise is strongly dependent on which pathway ABA is more sensitive to. The behaviour of noise as *β* is varied thus reflects two aspects of the two-pathway system above: when *Λ*_*d*_ > *Λ*_*s*_, increasing *β* enhances the dominance of the degradation pathways, and when *Λ*_*s*_ > *Λ*_*d*_, increasing *β* enhances the dominance of the synthesis pathway.

At higher *Λ*_*s*_, increasing *β* intuitively increases *ϕ*_*A*_ and decreases noise; at higher *Λ*_*d*_, increasing *β* decreases *ϕ*_*A*_ and increases noise, leading to two regions of high *η* (low *Λ*_*s*_, high *β*; and high *Λ*_*s*_, low *β*). In the case of high *Λ*_*s*_ and low *β*, scaled noise *η*′ can increase substantially. Low *β* and high *Λ*_*d*_ also induces a high *η*′, which can be understood through an enhanced influence of the intermediate *X* which itself is highly variable due to a comparatively low expression level.

The behaviour of *ϕ*_*A*_ and *η* with *Λ*_*s*_ and *Λ*_*d*_ is intuitive, with more synthesis leading to higher hormone levels and lower noise, and more degradation leading to lower hormone levels and higher noise. However, the relative strength of these influences leads to complex behaviour in scaled noise *η*′, which shows two peaks at intermediate *Λ*_*s*_ for low *Λ*_*d*_, and at low *Λ*_*s*_ and high *Λ*_*d*_. The structure of this (*Λ*_*s*_, *Λ*_*d*_) behaviour is similar to that in the direct feedback system, with the addition of the high-*Λ*_*d*_ peak in *η*′ due to the stronger influence of *Λ*_*d*_ on decreasing *ϕ*_*A*_.

The magnitudes of noise, and particularly scaled noise *η*′, in the single-pathway system are usually lower than in the two-pathway system above, reflecting the increased ability of two independent stochastic pathways to induce noise in the underlying hormone levels. The bimodal behaviour in *η*′ observed in the single-pathway system is often of low magnitude compared to the stronger trends observed in the case of two antagonistic pathways.

## 3. Discussion

We have used stochastic modelling to investigate the behaviour of a regulatory motif, recently identified in plant cells [37], that acts as a ‘random output generator’ governing the levels of ABA in seeds. The key feature of this motif is its positive feedback regulation of both ABA synthesis and degradation pathways, allowing the maintenance of constant mean ABA levels in concert with tunable control of ABA variability. ABA governs germination propensity (through an antagonistic relationship with another hormone GA [38,39]); variability in ABA therefore translates into variation in germination propensity [25,37]. The system we investigate is capable of generating tunable levels of noise in hormone levels while preserving mean levels, hence allowing plants to naturally vary germination propensity and allowing an evolutionarily beneficial bet-hedging strategy against varying environments [25,26,28]. Both genetic perturbations and differences in pathway activity can be used to modulate the levels of noise induced through this motif, providing the plant with a means to produce seeds of highly variable germination propensities, and a means for a population to navigate and adapt to selective pressures arising from varying environments. This variability is observed in germination experiments [43] (see above) and at the level of hormone abundance in observations of three-fold differences in ABA between seeds from the same silique [31]. We identify several routes for artificially adapting this cellular system to reduce this generation of variability, allowing for more uniform germination propensity when environments are less dynamic (as in agricultural contexts).

Among the findings of this stochastic modelling approach are the following: (a) symmetric modulation of synthesis and degradation pathways allows noise to be induced while preserving mean expression levels; (b) simple manipulations of the interactions within this regulatory system can be used to control hormone levels and noise in any combination of directions; (c) the limited experimental data currently available support our stochastic modelling of the features modulating ABA levels in *Arabidopsis*; (d) a symmetric two-pathway system with equal reaction rates is required for symmetric, robust control of noise while preserving expression levels; and (e) perturbations to simpler pathways can be identified to modulate noise and expression levels in a more restricted palette of options.

We underline that mathematical modelling with tools from stochastic processes is a powerful approach to explore questions associated with variability in cell biology. Biological variability is often challenging to experimentally characterize, requiring large numbers of observations and decoupling confounding sources of noise (including experimental uncertainty). Stochastic modelling affords the opportunity to make biological advances based on a bottom-up description of the system of interest, and can often be connected with what limited experimental evidence is available. In particular, the linear noise approximation we employ yields analytic expressions that can be explored in depth without necessitating time-consuming and less generalizable stochastic simulation; our previous work has also demonstrated the propensity of interpretable, powerful and simple analytic expressions to emerge from this treatment and drive scientific advances [44].

A natural follow-up question is how to quantitatively parametrize this system to model a given real plant. We first note that this study's qualitative predictions are perhaps its most important deliverable—the direction and relative magnitudes of effects that can be achieved by perturbing aspects of the system. The coarse-grained representation we employ necessarily omits some quantitative detail (for example, subtleties of stochastic gene expression, the full set of biochemical agents in each signalling pathway and the functional form of biochemical responses). Direct measurements of hormone levels are limited at this time (though some [43] provide support for our model as above), but readouts of relative protein abundance are available at a cellular resolution [37]. Moreover, experiments where ABA levels are increased in a bath of known concentration can be performed [37]. A combination of these readouts with parametric inference tools for stochastic biology [45] will allow further quantitative refinement of this modelling approach.

We have largely considered variability at the cellular level. In linking this approach to whole-seed behaviour, we must address the possibility that noise in individual cells is somehow ‘averaged out’ and is less important at the seed level. Several findings suggest that this picture may not be accurate. Existing work has shown that observed germination behaviour can be recapitulated by a model considering only a reduced subset of cells [37], suggesting a picture where the germination influence of a small number of cells may be amplified. Concurrently, the idea of a ‘threshold’ switch is widely used in considering germination [25,39,40,46,47]. A plausible mechanism giving rise to such threshold-like behaviour would involve a collective decision being reached, for example, when a given proportion of fluctuating cells exceed a threshold at the same time. In both these cases, the cell-to-cell variability in hormone levels would be crucial in governing germination, and levels of cell-to-cell variability are directly linked to germination variability. This picture is supported by the agreement between the predictions of our cell-level model and the limited seed-level statistics available [37,43]; further work taking a multiscale approach will be valuable in elucidating this link.

In our model, we have used a fixed set of initial conditions for each element of the system. However, the unique history of a given plant could contribute additional variability to the system, for example if variability in ABA levels in the previous generation is transmitted to the current generation. Our model contains a characteristic time scale with which initial states are remembered, but this quantity remains challenging to parametrize with existing data. The lack of empirical information on this time scale is a reason that we currently largely focus on the steady-state behaviour of moments in our model. Further experimental characterization of correlations in these cellular variables will enable future work to identify the time scales over which such memory contributes to the system's behaviour.

Our predictions of most importance for crop engineering, reflected by a combination of equations (2.12) and (2.13), are that modulating sensitivity *Λ*, response *β*, and the degradation rate of signalling intermediates *δ* will decrease noise in hormone abundance. This outcome is desirable in instances where variability in germination propensity is an agronomic issue, for example, in preventing uniform field crop establishment [25]. As previously discussed, seed variability is an evolutionarily beneficial trait in plants, allowing hedging against environmental change, but human control over crop plant environments means that this evolutionary priority takes lower precedence and this variability is therefore a source of inefficiency. Crop breeding is likely to have reduced some sources of variability, but the confounding involvement of these cellular actors in other processes of agronomic importance presents a limiting factor for previous strategies. We believe that detailed elucidation of the specific role that these actors play in noise generation will motivate new breeding strategies that reduce variability while limiting impact on these other traits. Synthetic perturbations to the genes involved in these signalling pathways represents a promising avenue for further engineering out these evolutionarily beneficial, but agronomically challenging, noise-generating mechanisms. We hope that this work, characterizing the noise-inducing behaviour of a regulatory motif central to germination in *Arabidopsis*, illustrates that stochastic modelling can identify targets for future genetic manipulation to lower seed variability and address consequent issues in crop establishment and food security.

## Supplementary Material

Supplementary Information

## Data accessibility

This article does not make direct use of new primary data; sources are given for all data used.
