# Supplementary material for: Identification of a bet-hedging network motif generating noise in hormone concentrations and germination propensity in Arabidopsis
Source: J R Soc Interface. 2018 Apr 11;15(141):20180042. doi: 10.1098/rsif.2018.0042 (PMC5938590; doi:10.1098/rsif.2018.0042)
Supplement: Supplementary Information [file rsif20180042supp1.pdf]

Supplementary Information: Identification of a bet-hedging network motif generating noise in hormone concentrations and germination propensity in Arabidopsis

Iain G. Johnston* and George W. Bassel
School of Biosciences, University of Birmingham, Birmingham, UK

(Published in J Roy Soc Interf)

Fluorescence microscopy.

The fluorescent reporter lines used are *AAO3::AAO3-GUS* [1], corresponding to the final step in ABA synthesis, and *CYP707A2::CYP707A2-GUS* [2], corresponding to a limiting step in ABA degradation. Images were acquired using primary dormant embryos as described in Ref. [2] using an inverted Zeiss LSM710 laser-scanning confocal microscope. Processing was performed using MorphoGraphX [3].

System size expansion.

Given stoichiometric matrix S and a vector of rates $\mathbf{f}$, collecting terms in the system size expansion gives us

$$\frac{\partial \phi_i(t)}{\partial t} = \sum_{j=1}^{R} S_{ij} f_j, \tag{1}$$

describing the deterministic rate equations for the system, and

$$\frac{\partial \Pi(\xi, t)}{\partial t} = \sum_{j=1}^{R} \left(-\sum_{ik} S_{ik} \frac{\partial f_j}{\partial \phi_k} \frac{\partial \xi_k \Pi}{d\xi_i} + \frac{1}{2} f_j \sum_{ik} S_{ij} S_{kj} \frac{\partial^2 \Pi}{\partial \xi_i \xi_k} \right), \tag{2}$$

a Fokker-Planck equation describing the time evolution of the distribution $\Pi(\xi, t)$ of the fluctuating components.

For the symmetric system, the ODEs governing mean behaviour, arising from Eqn. 1, are:

$$\begin{aligned} d\phi_A/dt &= \lambda(1 + \Lambda\phi_S) - \nu(1 + \Lambda\phi_D)\phi_A & (3)\\ d\phi_S/dt &= \beta\phi_A - \delta\phi_S & (4)\\ d\phi_D/dt &= \beta\phi_A - \delta\phi_D. & (5) \end{aligned}$$

The steady-state solution of these equations then gives our predicted mean values.

Multiplying Eqn. 2 through by the appropriate powers of our variables and integrating, we obtain ODEs governing variances and covariances for the symmetric system:

$$\begin{aligned} d\langle\xi_A^2\rangle/dt &= \lambda + \lambda\Lambda\phi_S + \nu\phi_A(1 + \Lambda\phi_D - 2\Lambda\langle\xi_A\xi_D\rangle) + 2\lambda\Lambda\langle\xi_A\xi_S\rangle - 2\nu\langle\xi_A^2\rangle - 2\Lambda\nu\phi_D\langle\xi_A^2\rangle & (6)\\ d\langle\xi_D^2\rangle/dt &= \delta\phi_D + \beta\phi_A - 2\delta\langle\xi_D^2\rangle + 2\beta\langle\xi_A\xi_D\rangle & (7)\\ d\langle\xi_S^2\rangle/dt &= \delta\phi_S + \beta\phi_A - 2\delta\langle\xi_S^2\rangle + 2\beta\langle\xi_A\xi_S\rangle & (8)\\ d\langle\xi_A\xi_D\rangle/dt &= -\Lambda\nu\phi_A\langle\xi_D^2\rangle + \lambda\Lambda\langle\xi_S\xi_D\rangle - \delta\langle\xi_A\xi_D\rangle - \nu(1 + \Lambda\phi_D)\langle\xi_A\xi_D\rangle + \beta\langle\xi_A^2\rangle & (9)\\ d\langle\xi_A\xi_S\rangle/dt &= -\Lambda\nu\phi_A\langle\xi_S\xi_D\rangle + \lambda\Lambda\langle\xi_S^2\rangle - \delta\langle\xi_A\xi_S\rangle - \nu(1 + \Lambda\phi_D)\langle\xi_A\xi_S\rangle + \beta\langle\xi_A^2\rangle & (10)\\ d\langle\xi_S\xi_D\rangle/dt &= -2\delta\langle\xi_S\xi_D\rangle + \beta(\langle\xi_A\xi_D\rangle + \langle\xi_A\xi_S\rangle). & (11) \end{aligned}$$

Again, the steady-state solution of these equations gives our predictions for the variances and covariances, where $\langle\xi_A^2\rangle$ is of particular interest.

The expression for $\langle\xi_A^2\rangle$ in the single-pathway model, and for $\langle\xi_A^2\rangle$ in the two-pathway model when $\beta_s = \beta_d$, both have the form

---

*Electronic address: i.johnston.1@bham.ac.uk

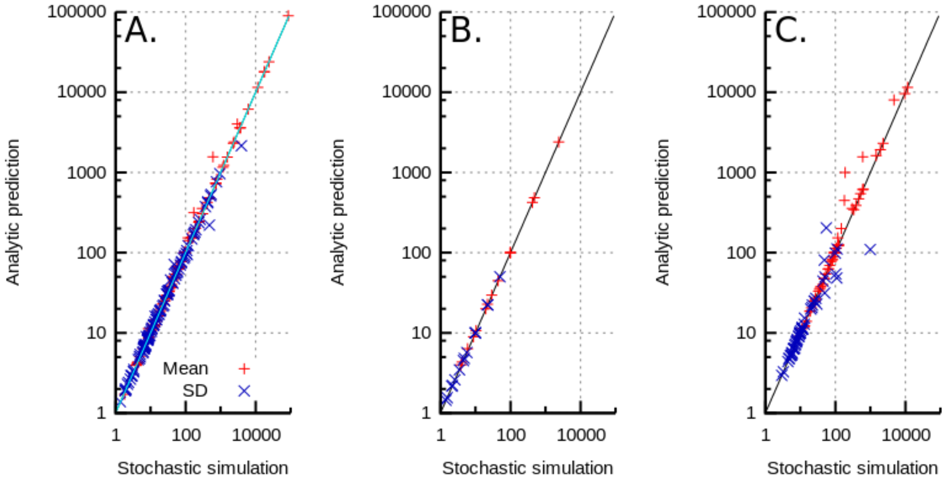

FIG. S1: **Comparing theory and simulation.** Values of ϕ_A and $\sqrt{\langle \xi_A^2 \rangle}$ predicted from stochastic theory and emerging from stochastic simulation, for a set of parameterisations of each model in the main text that spans the phase space portraits Figs. **??** and **??**. The strong agreements supports the validity of the linear noise approximation throughout this investigation.

$$\langle \xi_A^2 \rangle = \frac{\beta\lambda^2\Lambda_s(1+\phi_A(\beta-2)\Lambda_s) - \phi_A\nu(1+\nu+\phi_A\beta\Lambda_d(1+(3+2\phi_A(\beta+1)\Lambda_d)\nu)) - \lambda(1+\nu+\phi_A\beta(\Lambda_s+\Lambda_d(2+\phi_A\gamma\Lambda_s)\nu))}{2(\beta\lambda\Lambda_s - \nu - 2\phi_A\beta\Lambda_d\nu)(1+\nu+\phi_A\beta\Lambda_d\nu)} \quad (12)$$

For the two-pathway case, $\gamma \equiv \beta$; for the one-pathway case, $\gamma \equiv (\beta - 4)$.

Theory-simulation comparison.

Fig. S1 shows the agreement between theory and stochastic simulation for parameterisations and models used throughout this study.

Germination propensities.

The experimental data from Ref. [4] takes two forms. First, several replicates of the following experiment were performed. Two sets of 30 seeds of wildtype plants and two sets of 30 seeds of *pTaEM:SbNCED* mutant plants (increasing ABA levels) are plated. The germination proportion in each set of 30 seeds is recorded after 5 days, obtaining proportions $p_{WT,1}, p_{WT,2}, p_{MU,1}, p_{MU,2}$ respectively for the two wildtype (WT) and two mutant (MU) sets. We report the (untransformed) mean germination proportion for WT ($\mu_{WT} = p_{WT,1} + p_{WT,2}$ and MU ($\mu_{MU} = p_{MU,1} + p_{MU,2}$). Next we logit transform the individual germination proportions $p_{\circ,\circ}$ using $p'_{\circ,\circ} = \log(p_{\circ,\circ}/(1-p_{\circ,\circ}))$. We then take the (transformed) standard deviation of WT and MU ($\sigma'^2_\circ = \sum_i (p'_{\circ,i} - \mu_\circ)^2$). The statistics we plot are $(\mu_{WT}, \sigma'_{WT}), (\mu_{MU}, \sigma'_{MU})$.

Second, several replicates of the following experiment were performed. ABA levels are measured in 3 sets of 50 wildtype seeds and 3 sets of 50 *pTaEM:SbNCED* mutant seeds. The mean μ and standard deviation σ of ABA mass per seed are reported. The statistics we plot are μ and the coefficient of variation $\eta = \sigma/\mu$.

Dependence on degradation parameters.

In the main text we set $\delta_s = \delta_d = 1$ to allow an intuitive exploration of the system's behaviour. Fig. S2 shows some sampled effects of relaxing this assumption and allowing δ_s and δ_d to vary independently over orders of magnitude. While a quantitatively diverse range of behaviours is exhibited, an intuitive general trend is seen. Decreasing δ_d increases the influence of the ABA degradation pathway, lowering mean ABA levels and increasing noise. Decreasing δ_s has the opposite effect, increasing the influence of the synthesis pathway, increasing mean ABA levels and decreasing noise. Increasing δ_d or δ_s has a comparatively limited effect in many cases; as the other parameter values are constrained to lie on $[0, 1]$, excess degradation of one pathway has limited influence once this pathway provides only a weak contribution to the overall dynamics.

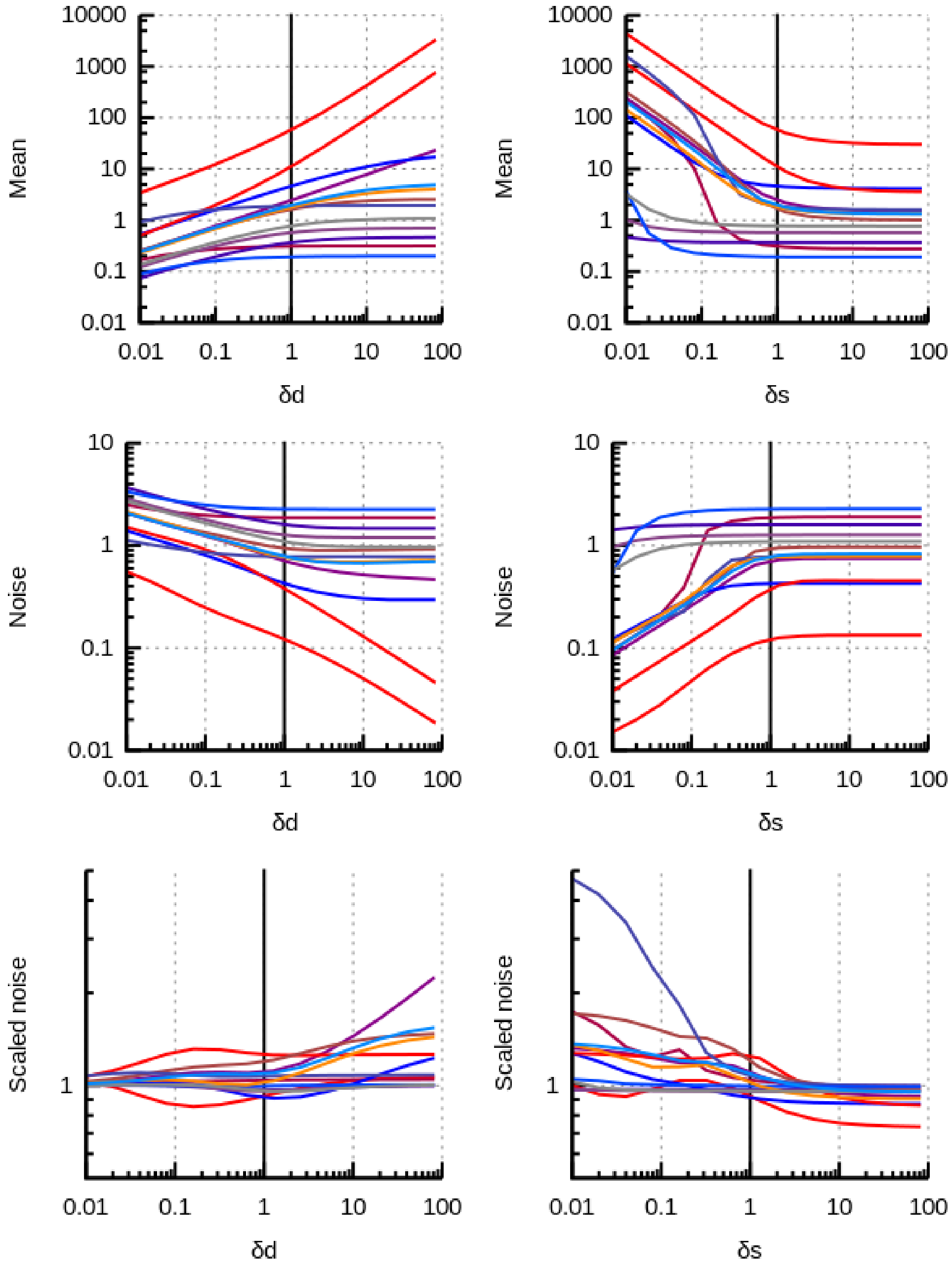

FIG. S2: **Sampled effect of varying degradation parameters.** For each trace, the values of $\beta_s, \beta_d, \nu, \lambda, \Lambda_s, \Lambda_d$ are randomly sampled from $\mathcal{U}(0,1)$. One of δ_d, δ_s is fixed at 1 and the other is varied over orders of magnitude (horizontal axis). Steady-state ABA mean, noise, and scaled noise are then reported (vertical axis).

[1] Mitsunori Seo, Atsushi Hanada, Ayuko Kuwahara, Akira Endo, Masanori Okamoto, Yukika Yamauchi, Helen North, Annie Marion-Poll, Tai-ping Sun, Tomokazu Koshiba, et al. Regulation of hormone metabolism in *Arabidopsis* seeds: phytochrome regulation of abscisic acid metabolism and abscisic acid regulation of gibberellin metabolism. *The Plant Journal*, 48(3):354–366, 2006.

[2] Alexander T Topham, Rachel E Taylor, Dawei Yan, Eiji Nambara, Iain G Johnston, and George W Bassel. Temperature variability is integrated by a spatially embedded decision-making center to break dormancy in *Arabidopsis* seeds. *Proceedings of the National Academy of Sciences*, page 201704745, 2017.

[3] Pierre Barbier de Reuille, Anne-Lise Routier-Kierzkowska, Daniel Kierzkowski, George W Bassel, Thierry Schüpbach, Gerardo Tauriello, Namrata Bajpai, Sören Strauss, Alain Weber, Annamaria Kiss, et al. Morphographx: a platform for quantifying morphogenesis in 4d. *Elife*, 4:e05864, 2015.

[4] Mariko Nonogaki, Khadidiatou Sall, Eiji Nambara, and Hiroyuki Nonogaki. Amplification of ABA biosynthesis and signaling through a positive feedback mechanism in seeds. *The Plant Journal*, 78(3):527–539, 2014.
